# Retinal microstructual and microvascular changes in mucopolysaccharidoses

**DOI:** 10.1007/s00417-025-06954-y

**Published:** 2025-10-18

**Authors:** Lucas Wipprecht, Anna J. Borchers, Julia V. Stingl, Lucia Segura Schmitz, Karoline von Plettenberg, Julia B. Hennermann, Susanne Hopf, Susanne Pitz, Juliane Matlach

**Affiliations:** 1 Department of Ophthalmology, University Medical Center of the Johannes Gutenberg-University of Mainz, Mainz, Germany; 2 Center for Pediatric and Adolescent Medicine, Villa Metabolica, University Medical Center of the Johannes Gutenberg-University of Mainz, Mainz, Germany; 3 Department of Ophthalmology, Bürgerhospital Frankfurt, Orbitazentrum, Frankfurt, Germany; 4 Department of Ophthalmology, University of Leipzig, Leipzig, Germany; 5 Department of Ophthalmology, University Medical Center of the Johannes Gutenberg-University Mainz, Semmelweisweg 120, D-55131 Mainz, Germany

**Keywords:** Mucopolysaccharidoses, Retinopathy, Optical coherence tomography, Optical coherence tomography angiography, High magnification module

## Abstract

**Purpose:**

To analyse ocular manifestations of MPS in the posterior segment of the eye, in particular retinal and optic disc pathologies, compared to healthy controls.

**Methods:**

This prospective study analyzed structural and functional posterior eye changes in 29 MPS patients (58 eyes) compared to 29 healthy, age- and gender-matched controls. Examinations included visual acuity testing, orthoptic status, intraocular pressure (IOP) measurement, visual field testing, slit-lamp examination and fundus examination, Spectral-Domain Optical Coherence Tomography (SD-OCT), OCT-Angiography (OCT-A) and High Magnification Module^®^ (HMM^®^) imaging.

**Results:**

LogMAR visual acuity (*p* < 0.001) and IOP (*p* < 0.001) were significantly worse/higher in MPS patients. Visual field defects showed a concentric restriction pattern, resembling to pigmentary retinopathy. Fundus examination of 56 eyes revealed optic disc atrophy in two eyes (all MPS II), atrophic macula in two eyes (both MPS II) and pigmentary retinopathy in twelve eyes (6 MPS II, 6 MPS IV). Total retinal thickness was significantly reduced in the parafoveal (*p* < 0.001) and perifoveal (*p* < 0.001) macula area in MPS, especially in MPS II patients. Peripapillary retinal nerve fiber layer thickness correlated positively with IOP (*p* = 0,015) in the MPS group. OCT-A findings revealed reduced capillary density of the parafoveal and perifoveal retina in 8/22 eyes in the MPS group (2 MPS I, 4 MPS II, 2 MPS IV). Rarefaction of the photoreceptor mosaic was observed in some MPS patients on HMM imaging.

**Conclusion:**

MPS patients exhibit impaired visual acuity and visual fields, higher IOP, and mircostructural and microvascular alterations of the optic disc and retina compared to healthy subjects.

## Key messages

*****What is known?***:**

- Depending on the subtype, MPS patients show different pathological changes in the posterior segment of the eye, including pigmentary retinopathy, and optic disc edema or atrophy.

*****What is new?***:**

- Both funduscopy and imaging revealed changes in the posterior segment of the eye, particularly in MPS type II, with significant thinning of the retina in the parafoveal and perifoveal regions.
- For the first time, quantitative OCT images were obtained in MPS III patients, who have the most severe form of retinopathy according to literature, providing a basis for further studies.
- In several MPS subtypes, OCT angiography showed a reduced capillary density and HMM images demonstrated a reduction of cone photoreceptors.

## Introduction

Mucopolysaccharidoses (MPS) are a group of lysosomal storage disorders caused by the absence or deficiency of different lysosomal enzymes [1]. As a result, partially degraded glycosaminoglycans (GAG) accumulate in various tissues, leading to short stature, facial dysmorphism, hepatosplenomegaly, partially neurological impairment, and occular changes. To date, 8 types of MPS have been identified, each caused by different enzyme deficiencies. The prevalence varies by region and ethnicity, with an estimated 3.51 cases per 100,000 individuals in Germany [2]. 

Ocular changes in MPS patients include vision loss, amblyopia, mostly hyperopia and astigmatism, stromal corneal clouding, increased intraocular pressure (IOP), strabismus, pigmentary retinopathy, and optic disc edema or atrophy [3–8]. 

While various studies evaluated the severity and frequency of ocular manifestations in different MPS types, only a few analyzed structural changes in the posterior eye.(3–5).

Diagnosing posterior segment pathologies in MPS is challenging, particularly in patients with severe corneal opacification, which can make visibility of the posterior segment difficult [9]. The types MPS III and VI are often underrepresented in ophthalmological studies. Furthermore, only one recommendation for general ophthalmic examinations of MPS patients has been published [7]. 

In this study, we utilized advanced imaging techniques to characterize and quantify ocular alterations in the posterior segment of MPS patients compared to healthy subjects.

## Materials and methods

### Patients

This clinical comparative prospective study was performed between April 2021 and October 2022 at the University Medical Center Mainz, Germany. MPS patients were recruited from the Villa Metabolica, Center for Pediatric and Adolescent Medicine at the Mainz University Medical Centre, while healthy age- and gender-matched volunteers were recruited from the pediatric and ophthalmology departments of the University Medical Center Mainz. All examinations took place at the Department of Ophthalmology at the Mainz University Medical Centre. The study was approved by the Medical Ethics Committee of the State Chamber of Medicine of Rhineland-Palatinate in Mainz, Germany (reference number 2021–15589) and adhered to the tenets of the Declaration of Helsinki. Informed consent was obtained from all participants or their legally authorized representatives prior to enrollment.

To ensure reliability of examinations, only patients aged 6 years and older were enrolled in the study. The MPS group included individuals with enzymatically and/or genetically confirmed diagnosis of MPS. Healthy subjects with a spherical equivalent refractive error less than − 3.0 diopters and greater than + 3.0 diopters and an astigmatism refractive error less than − 2.0 diopters and greater + 2.0 diopters were excluded. Inclusion criterion for healthy subjects was no history of ocular pathology or surgery. Based on the above criteria, a maximum of 29 patients with MPS were recruited.

### Ocular examinations

All patients received a full orthoptic and ophthalmologic examination including best-corrected visual acuity testing with Landolt ring or Cardiff Cards depending on cognitive abilities, stereo-optical testing, refraction with auto refractometer OCULUS AR-1s (OCULUS optic devices GmbH, Wetzlar, Germany) or PLUSOPTIX A09 (Plusoptix GmbH, Nürnberg, Germany), applanation tonometry or palpatory measurement of IOP, slit-lamp examination for grading corneal clouding [7] and dilated fundus examination using tropicamid eye drops.

A logMAR greater than 0.4 was defined suspicious for amblyopia or vision loss caused by anterior or posterior segment abnormalities. Myopic, hyperopic and astigmatic refractive anomalies were defined as any deviation in refraction. An IOP above 20mmHg was considered a potential risk factor for the presence of glaucoma. IOP was adjusted using the Dresdner correction formula.

Central 30°-visual fields were assessed using the OCTOPUS 900 (HAAG-STREIT Germany GmbH, Wedel, Germany). The mean defect (MD), representing the average of all measured visual field defects, and the square root loss of variance (sLV), which differentiates between local and diffuse defects, were recorded. An MD value exceeding 10 dB was classified as a severe visual field defect. Pachymetry was recorded using the OCULUS Pentacam^®^ HR (OCULUS Optikgeräte GmbH, Wetzlar, Germany).

Horizontal OCT scans centered on the fovea were performed using the Spectralis SD-OCT (Heidelberg Engineering GmbH, Heidelberg, Germany), operating at 40,000 A-scans per second with an axial resolution of 7 μm. Immediately after acquisition, image quality and usability were reviewed. All scans were conducted by 2 qualified orthoptists using the Spectralis Glaucoma Module. Macular scans were performed in Posterior Pole mode and segmented into the central fovea (1 mm diameter), parafoveal (3 mm diameter), and perifoveal (6 mm diameter) regions [10]. Total retinal thickness in these areas was measured. Peripapillary retinal nerve fiber layer (pRNFL) thickness was assessed using circular peripapillary scans with diameters of 3.5, 4.1, and 4.7 mm [11]. Additionally, OCT-A was performed of the macula, and in-vivo visualization of photoreceptors was performed with the High Magnification Module^®^ (HMM^®^). While the HMM^®^ imaging method does not produce reproducible images, hyporeflective areas in the HMM^®^ scans corresponded to notable photoreceptor reduction.

### Statistical analyses

All statistical analyses were performed using SPSS (version 27.0, IBM, Chicago, Illinois, USA). Generated data were expressed with median and interquartile range (IQR) for non-normally distributed variables, with non-parametric Mann-Whitney U tests applied for group comparisons. Correlation analyses (Pearson and Spearman tests) were conducted to assess relationships between functional and structural findings. Given the exploratory nature of the study, no adjustments were made for multiple testing, and p-values were interpreted descriptively.

## Results

### Patient demographics and ocular characteristics

In our study, 29 MPS patients (58 eyes) - two with MPS I (1 MPS H, 1 MPS HS), eleven with MPS II, six with MPS III (5 MPS IIIA, 1 MPS IIIB), seven with MPS IV (all MPS IVA) and three with MPS VI - and 29 healthy controls (58 eyes) were included. Mean age of the MPS group was 20 years (IQR 18 years) and included eight (28%) female and 21 (72%) male patients. Mean age of the healthy control group was 22 years (IQR 20 years) with 19 (66%) female and 10 (35%) male participants. The ocular characteristics of the MPS types and the healthy participants are shown in Table 1.

**Table 1:** Ocular characteristics of mucopolysaccharidoses (MPS) patients and healthy subjects

		MPS I		MPS II	MPS III		MPS IV	MPS VI	Healthy subjects
Subtype		H	HS		A	B	A		
Gender	Female/male	1/0 (*n* = 1)	0/1 (*n* = 1)	0/11 (*n* = 11)	2/3 (*n* = 5)	0/1 (*n* = 1)	4/3 (*n* = 7)	1/2 (*n* = 3)	19/10 (*n* = 29)
Age		18 (18)	47 (47)	25 (30)	12 (9)	37 (37)	20 (13)	29 (15)	22 (20)
HSCT		1 (100%)	0	0	0	0	0	0	0
ERT		0	1 (100%)	10 (90%)	0	0	4 (57%)	2 (67%)	0
Antiglaucoma medication		1 (100%)	0	0	0	0	0	1 (33%)	0
Eye surgery		0	1 (100%)	1 (9%)	0	0	0	1 (33%)	0
Binocular vision		1 (100%)	0	9 (82%)	2 (40%)	1 (100%)	7 (100%)	2 (67%)	29 (100%)
Corneal clouding	Mild	0	2 (100%)	6 (27%)	0	0	5 (36%)	0	0
	Moderate	2 (100%)	0	0	0	0	7 (50%)	2 (33%)	0
	Severe	0	0	0	0	0	0	2 (33%)	0
	Absent	0	0	16 (73%)	10 (100%)	2 (100%)	2 (14%)	2 (33%)	58 (100%)
Optic nerve	Normal	2 (100%)	1 (50%)	18 (82%)	8 (80%)	2 (100%)	12 (100%)	2 (33%)	58 (100%)
	Atrophy	0	0	4 (18%)	0	0	0	0	0
	Not visible	0	1 (50%)	0	2 (20%)	0	0	4 (67%)	0
Macula	Normal	2 (100%)	1 (50%)	18 (90%)	6 (60%)	2 (100%)	14 (100%)	2 (33%)	58 (100%)
	Atrophy	0	0	2 (10%)	0	0	0	0	0
	Not visible	0	1 (50%)	0	4 (40%)	0	0	4 (67%)	0
Retina	Normal	2 (50%)	0	14 (64%)	6 (60%)	2 (100%)	8 (57%)	2 (33%)	58 (100%)
	Pigmentary retinopathy	0	0	6 (27%)	0	0	6 (43%)	0	0
	Not visible	0	2 (50%)	0	4 (40%)	0	0	4 (67%)	0

Data are presented as absolute values/number of eyes (percentage), median (interquartile range) as appropriate.

*MPS* mucopolysaccharidoses, *HSCT* hematopoietic stem cell therapy, *ERT* enzyme replacement therapy

One MPS IH patient had received hematopoietic stem cell therapy, 17 patients (1 MPS IHS, 10 MPS II, 4 MPS IVA, 2 MPS VI) were on enzyme replacement therapy. Additionally, one MPS I patient underwent penetrating keratoplasty in the right eye, one MPS II patient underwent cataract surgery in both eyes, and one MPS VI patient had a decompression of the right optic nerve.

The logMAR visual acuity (Table 2) of the MPS group was significantly higher (*p* < 0.001) than that of the healthy participants. Among our patients, 65% had a logMAR visual acuity greater than 0.4, which can be suggestive of amblyopia or a retinal or optic nerve disease. In the visual acuity test, two eyes (one MPS I and one MPS VI) were only able to detect light movements and two other eyes (both MPS III) were only able to fixate and follow an object or light. In the MPS group, 55 (95%) eyes were measured with an autorefractometer. Among them, six (11%) were myopic, one (2%) was emmetropic, and 48 (87%) were hyperopic. Additionally, 53 (96%) eyes had an astigmatism. Spherical refraction ranged between − 1.0 diopters and + 17.0 diopters.

**Table 2:** Comparison of examination including optical coherence tomography (OCT) measurements between mucopolysaccharidoses (MPS) and healthy controls

	Healthy subjects	MPS	*P*-value
Visual acuity (logMAR)	−0.03 ± 0.12 (*n* = 58)	0.43 ± 0.62 (*n* = 58)	< 0.001
Intraocular pressure (mmHg)	12.75 ± 1.71 (*n* = 56)	17.23 ± 4.22 (*n* = 52)	< 0.001
Visual field (mean defect [dB])	1.46 ± 2.89 (*n* = 52)	11.13 ± 9.06 (*n* = 26)	< 0.001
Visual field (square root loss of variance [dB])	3.22 ± 1.39 (*n* = 52)	5.44 ± 2.06 (*n* = 26)	< 0.001
Central macular thickness (µm)	268.9 ± 14.66 (*n* = 58)	270.57 ± 60.93 (*n* = 42)	0.11
Parafoveal macular thickness (µm)	345.99 ± 12.47 (*n* = 58)	318.66 ± 49.68 (*n* = 43)	< 0.001
Perifoveal macular thickness (µm)	306.33 ± 14.16 (*n* = 58)	277.77 ± 40.24 (*n* = 43)	< 0.001
pRNFL (µm)	111.83 ± 11.48 (*n* = 56)	110.88 ± 20.52 (*n* = 33)	0.39

Data are presented as mean ± standard deviation, or median (interquartile range). Mann–Whitney U-test

*pRNFL* peripapillary retinal nerve fiber layer, *MPS* mucopolysaccharidoses, *OCT* Optical Coherence Tomography

### IOP and visual field (Table 2)

IOP, both with and without adjustment for corneal thickness, was significantly higher (*p* < 0.001) in the overall MPS group, as well as in types III and IV, compared to healthy participants (adjusted: 16.0 mmHg vs. 12.8 mmHg; unadjusted: 17.2 mmHg vs. 12.8 mmHg). Corneal thickness did not differ significantly between the MPS group and the control group (637.28 μm vs. 547.07 μm; *p* = 0.82). Eight (14%) eyes of MPS patients had an IOP of more than 20 mmHg, however only two patients (one MPS I and two MPS VI eyes) were treated with antiglaucoma medication. The MPS group showed a non-significant positive correlation (*p* = 0.08) between IOP and logMAR visual acuity.

**Fig. 1:**
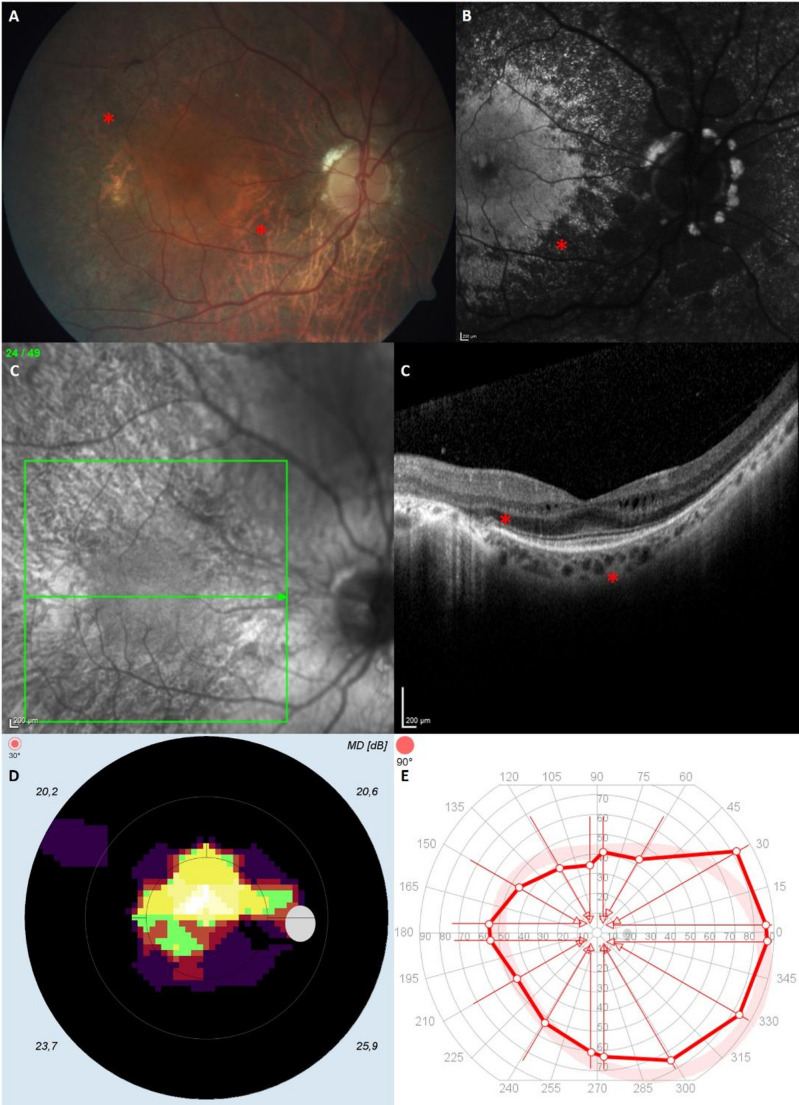
Degenerative pigmentary retinopathy in mucopolysaccharidosis (MPS). Case of a 46 year old MPS II patient: Fundus photography shows retinal atrophy and bone spicules (“pigmentary retinopathy”) (**A** marked with *), fundus autofluorescence with hypofluorescence in degenerative retinal areas (**B** marked with *), Optical Coherence Tomography (OCT, Fundus image left, retinal layers right) reveals perifoveal atrophy of the outer retinal layers and intraretinal cysts (**C** marked with *), concentric visual field restriction in the 30° perimetry (**D**) with normal outer boundaries in kinetic perimetry (optical test mark III 4e) (**E**)

Visual field MD and slV in the MPS group were significantly higher than in the healthy participants (both *p* < 0.001). Visual field defects in MPS affected larger retinal alreas and mostly showed a concentric restriction similar to pigmentary retinopathy. Visual field defects were more pronounced in those MPS patients with corneal opacities compared to the control group. Patients without corneal opacity showed a concentric narrowing of the visual fields, similar to retinitis pigmentosa, altough the kinetic visual fields were usually less severly restricet then the static visual fields (Fig. 1).

### Retinal and optic disc abnormalities

The typical retinal abnormalities in a MPS II patient are depicted in Fig. 1. Stromal corneal clouding is very rare in MPS II patients, while they typically present with degenerative pigmentary retinopathy accompanied by secondary optic atrophy, macular edema, and concentric visual field restriction without blindness (Fig. 1).

**Fig. 2:**
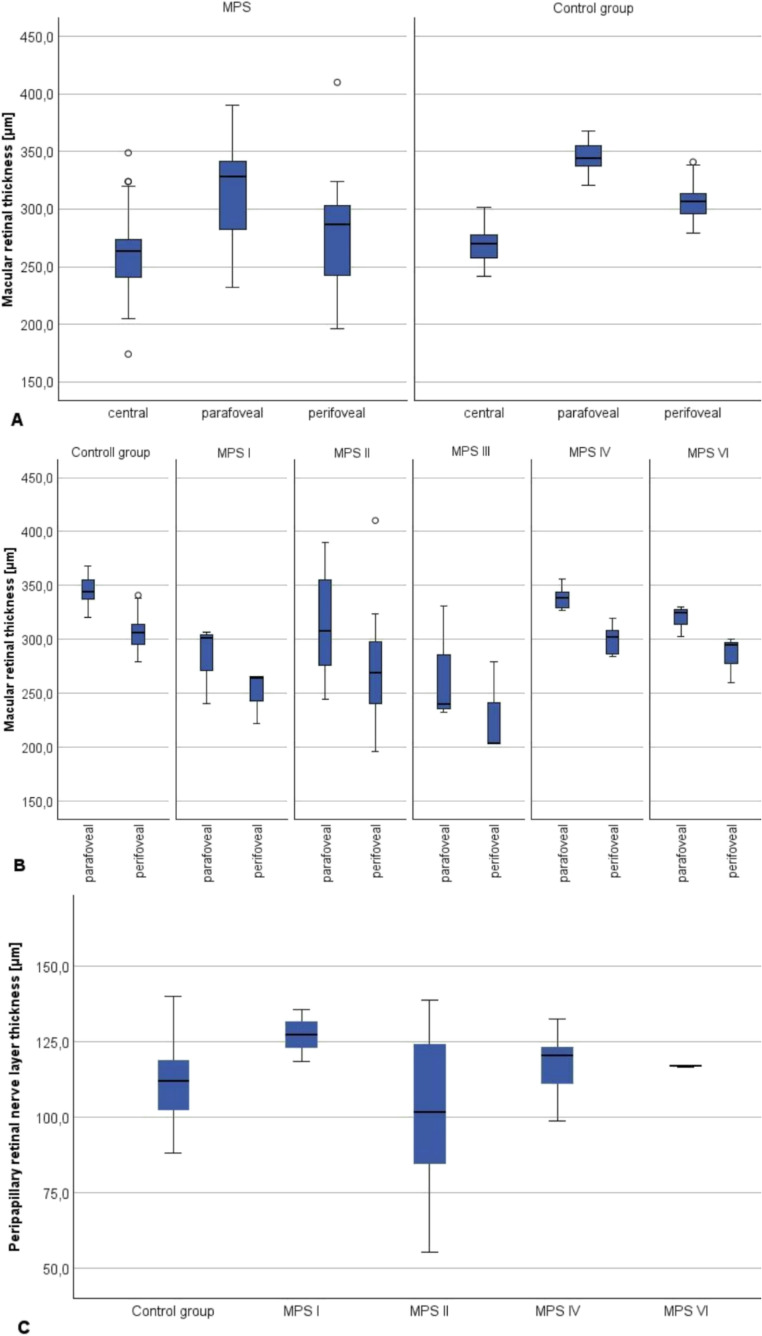
Macular retinal thickness and peripapillary retinal nerve layer thickness (pRNFL) on Optical Coherence Tomography (OCT) of the mucopolysaccharidoses (MPS) patients compared to the healthy participants. All MPS types showed a thinner parafoveal and perifoveal thickness compared to healthy participants (*p* < 0.05; **A**). Central retinal thickness did not show a statistically significant difference between MPS and the control group (**B**). The measured pRNFL thickness was slightly thinner in MPS patients than in the control group but did not show a statistical difference (*p* = 0.4; **C**) Box plots display the median (line inside the box) and the edges of the box mark the 25th quartile and 75th quartile with the interquartile range is between the 25th quartile and 75th quartile. Whiskers extend from the box are the 1.5 x interquartile ranges and any points outside this range considered as outliers (circles)

The results of the clinical examinations and OCT analyzed in the study are shown in Tables 1 and 2. Retinal thickness in parafoveal area was significantly thinner (*p* < 0.001) in the MPS group than in the healthy participants. Each MPS type, except MPS IV, had a thinner parafoveal retinal thickness than in the control group, but only in MPS II this difference was significant (*p* = 0.005; Fig. 2). Similarly, perifoveal retinal thickness was significantly reduced in the MPS group (*p* < 0.001), whereas in all MPS types, except MPS IV, perifoveal retinal thickness was thinner than in healthy participants, but without statistical significance. In MPS IV, retinal thickness in both parafoveal and perifoveal areas was nearly the same as in healthy participants. Total retinal thickness in the central fovea showed little difference between the MPS group and healthy participants (*p* = 0.11). The pRNFL thickness was slightly increased in MPS I, IV and VI, and tended to be thinner in MPS II than in healthy participants. MPS patients with funduscopic signs of optic neuropathy had a thinner peripapillary retinal nerve fiber layer (pRNFL; 75.1 μm) compared to the overall MPS and control groups, while parafoveal (348.0 μm) and perifoveal (324.9 μm) retinal thickness were increased. There was a positive correlation between pRNFL thickness and IOP in the MPS group (*p* = 0.015; Fig. 2).

**Fig. 3:**
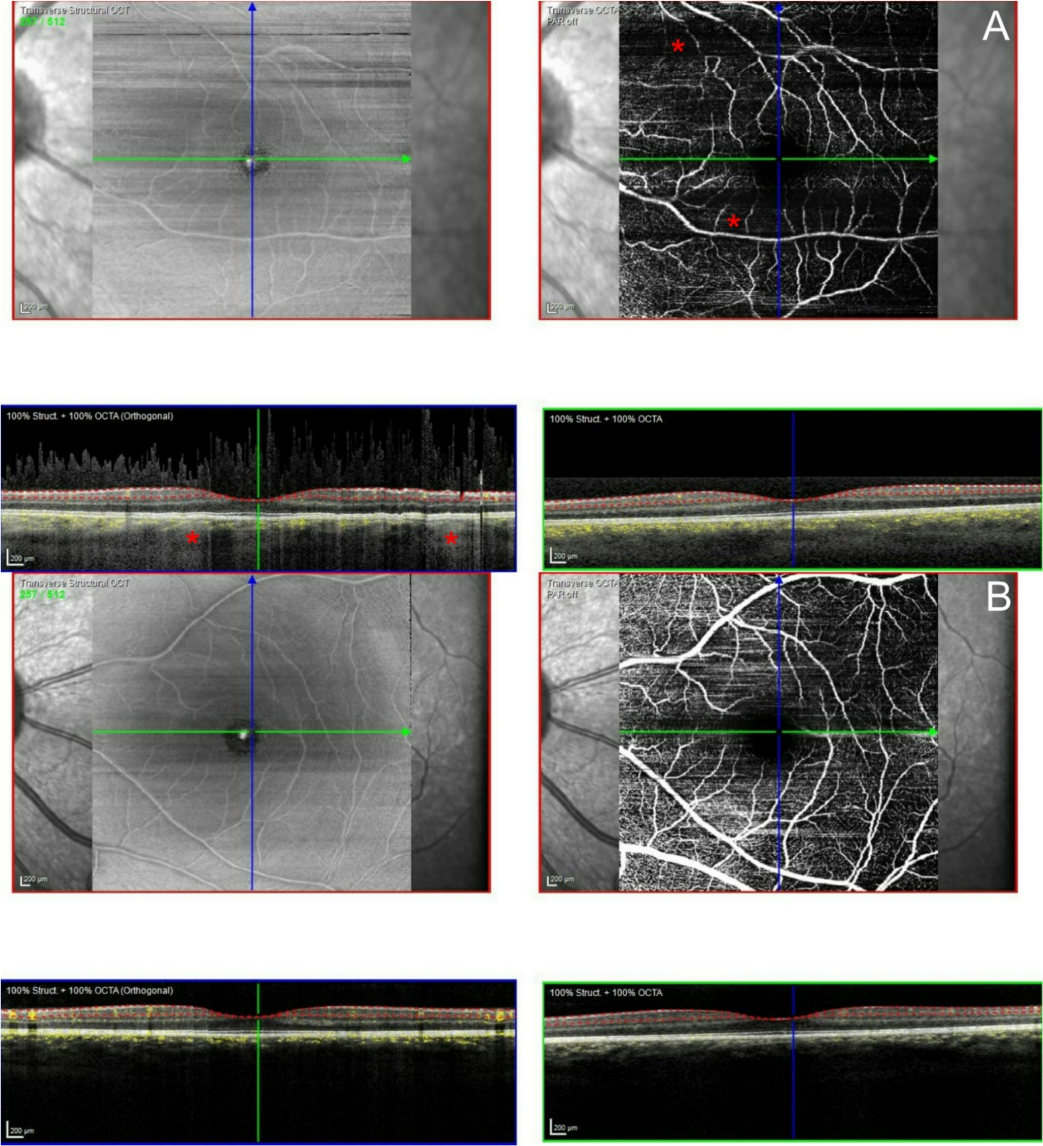
Macular Optical Coherence Tomography Angiography (OCT-A, Composite of 4 images; top left: Structural OCT, top reight: OCTA, bottom left: orthogonal OCTA slice, bottom right: transversal OCTA slice) of a mucopolysaccharidosis (MPS) patient and a healthy control. A case of a 24 year old MPS IVA patient with reduced capillary density (**A**, marked with *,) and a healthy partipant with normal capillary density (**B**)

OCT-A was performed in 22 (38%) eyes of the MPS group, with 8 (36%) eyes classified as abnormal due to a lower capillary density in the parafoveal and perifoveal retina (two MPS I, four MPS II, two MPS IV; Fig. 3).

**Fig. 4:**
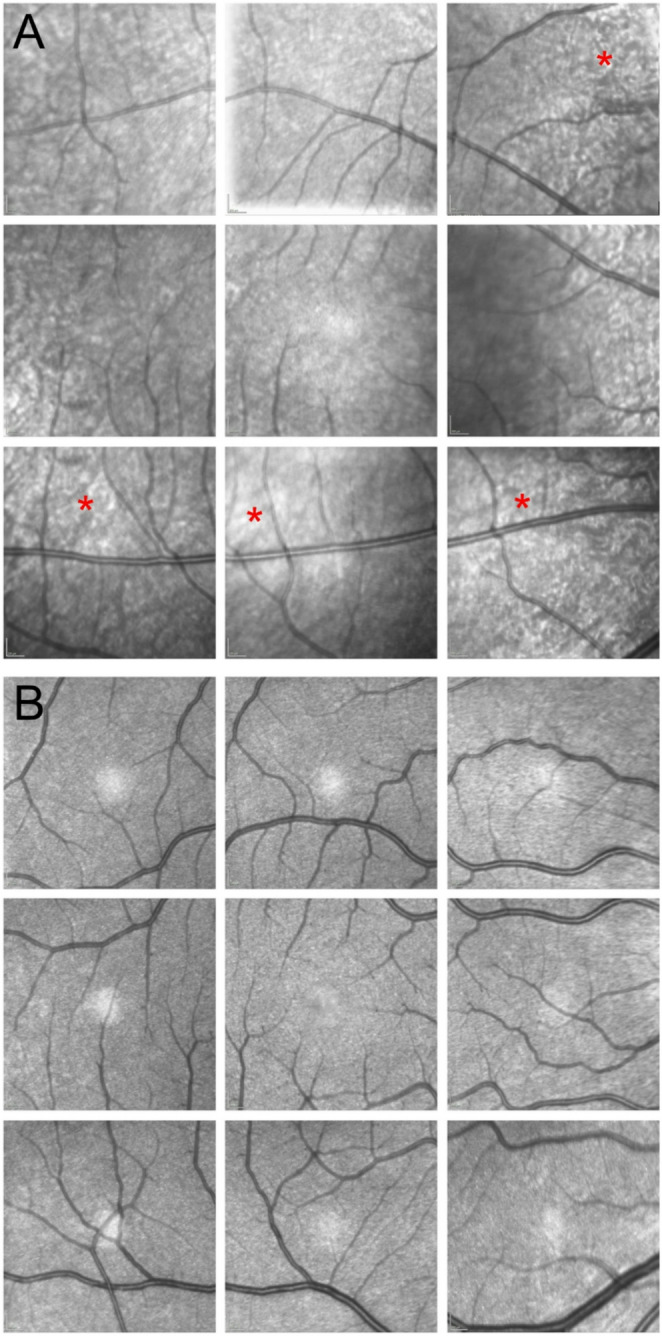
Cone photoreceptor imaging using High-Magnification-Module (HMM^®^) with Optical Coherence Tomograhy (OCT) in a mucopolysaccharidosis (MPS) patient and a healthy control in the right eye (consisting of 9 images). Case of a 22 year old MPS II patient with rarefaction of photoreceptors (white dots) in hyporeflective areas of the macula (**A**, exemplary marked with *) and a healthy participant with visible cone photoreceptors (**B**)

HMM^®^-imaging was performed in 19 (33%) eyes of the MPS group. A slight reduction of the photoreceptors, appearing as hyporeflective areas, was observed in some cases (Fig. 4). Image quality was too poor for analysis in 3 of 19 eyes.

## Discussion

This study describes and quantifies clinical ocular characteristics in patients of different MPS types, compared to age- and gender-matched healthy subjects. Due to the rarity of MPS, there are only a few studies, usually with a small number of cases, that describe typical ocular abnormalities [3–7]. To our knowledge, there has been no study yet using HMM^®^ imaging in MPS patients.

Although the primary aim of our study was to evaluate ophthalmological differences in MPS patients using advanced imaging, we recognize the importance of integrating genetic data to enhance genotype-phenotype correlations, particularly for ocular geneticists and pediatric retinal specialists. Specific gene mutations have been associated with disease severity, progression, and even particular ocular features such as corneal clouding or retinal degeneration. While our cohort lacked comprehensive molecular profiling, established genotype-phenotype studies offer valuable insights. In MPS I, Clarke et al. reported that homozygous or compound heterozygous loss-of-function IDUA mutations are typically associated with severe phenotypes, whereas attenuated forms often involve at least one missense or intronic variant [12]. In MPS II, Dvořáková et al. found that large IDS deletions or complex rearrangements strongly correlate with severe clinical presentations [13]. These findings were further supported by Muenzer et al., who analyzed data from over 650 patients in the Hunter Outcome Survey, showing that structural IDS mutations are consistently linked to cognitive impairment and more severe disease progression [14]. These studies underscore the value of incorporating detailed genetic data into future ophthalmic imaging studies, as this could help identify genotype-specific ocular biomarkers and support individualized monitoring strategies for MPS patients.

Previous studies revealed a significant reduction of visual acuity with a moderate to high prevalence of amblyopia or vision loss in MPS I, IV and VI and a mild prevalence in MPS II [3, 5, 9]. Our study yielded similar results. Notably MPS I, III and VI were associated with low visual acuity, with a mean logMAR over 0.4. Although 86% of MPS IV eyes had mild to moderate corneal clouding, they reached a mean visual acuity of 0.125 logMAR. In MPS, poorer binocular vision has been observed in cases with either severe corneal opacity or retinal pigmentary atrophy. It was not possible to differentiate between amblyopia due to corneal deprivation or orthoptic causes and poor visual acuity due to pathologies in the posterior segment of the eye such as pigmentary retinopathy. Therefore, we cannot confirm that the 65% of patients in our study with a logMAR over 0.4 had amblyopia. GAG accumulation likely leads to refractive errors such as hyperopia and astigmatism, as described in previous studies [4, 15, 16]. In our study, hyperopia and astigmatism were the most common refractive errors and were present in almost every MPS patient. Myopia was less common, but was more prevalent in MPS type III than in other MPS types, but this observation cannot be supported by other studies due to missing data.

Depending on the type of accumulated GAGs in the different MPS types, only certain parts of the eye are affected. A higher accumulation of dermatan sulfate causes more damage in the cornea, while a higher accumulation of heparan sulfate causes damage in the posterior segment of the eye, and accumulated keratan sulfate affects both parts [10, 17]. As a result, MPS types I, II and III are more likely to be affected by macular and retinal pigmentary atrophy [6, 7, 10]. In contrast to other studies, macular atrophy and retinal pigmentary atrophy only occurred in MPS II and IV in our study cohort [3, 18]. 

However, a measrued increased IOP is not necessarily associated with glaucoma in MPS. IOP measurements may be falsely elevated due to corneal thickening and stiffening caused by GAG deposition. To ensure a more accurate assessment of actual IOP, variations in corneal thickness should be taken into account, particularly in MPS patients with thicker corneal thickness [19]. The impact of corneal thickness and stiffness on IOP measurement depends on the method used [20]. Previous studies noticed that increased IOP could be present in every MPS type but is more severe in MPS I and VI [6]. Our study confirms previous findings that IOP can be elevated across all MPS types, both with and without adjustment for corneal thickness. However, we found higher percentage of eyes with increased IOP in MPS IV and VI patients. Dermatan sulfate and keratan sulfate are important components of the cornea and are discussed to regulate collagen fiber diameter and parallel arrangement of collagend fibrils [17]. Impairment of dermatan sulfate and keratan sulfate degradation may thus lead to corneal clouding and swelling and subsequently to glaucoma due to displacement of the chamber angle [15]. 

Visual field defects in MPS are thought to be related to retinopathy, optic disc atrophy, or glaucoma [6]. While Seok et al. reported varying degrees of restriction and enlarged blind spots in Goldmann kinetic perimetry in a previous study, no study has examined the effects of GAG accumulation on visual field defects using static perimetry [10]. Our study showed that performing a visual field examination is a valuable complementary diagnostic measure, particularly in patients without stromal opacity, to identify potential effects of GAG accumulation on the retina. Furthermore, static perimetry often shows more severe lmitations than kinetic perimetry. However, it is crucial to interpret the findings in the context of the ocular anatomy, the patient´s cognitive impairment and the available clinical findings.

In the current study, SD-OCT showed no significant difference in the central retinal thickness between MPS and healthy subjects, whereas other studies have shown an increased central retinal thickness caused by a thickened and hyperreflective external limiting membrane (ELM) [10, 21, 22]. In MPS II, central retinal thickness was only slightly higher than in healthy subjects in our study. The ELM consists of adherence junctions formed by photoreceptors of the inner segments and apical processes of the Müller cells. Müller cells are involved in retinal homeostasis and the functional regeneration of rod photoreceptors [23]. In MPS, GAG accumulation in Müller cells leads to functional changes and contributes to photoreceptor degeneration, resulting in ELM thickening [10]. This has only been observed in MPS I and II and was discussed as a consequence of heparan sulfate accumulation [1]. However, in our study, central retinal thickness was lower in MPS III and normal in MPS IV patients. Thus, heparan sulfate accumulation seems not to be the critical cause. In addition, Huang et al. described an irregular ELM by OCT in MPS VI patients not associated with heparan sulate storage [24]. 

In line with previously published studies, SD-OCT revealed significantly thinner parafoveal and perifoveal retinal thickness, particularly in MPS II, while retinal thickness in MPS IV was comparable to that of healthy subjects [10, 22]. Similar results were obtained in the sudy by Noor et al. who found retinal abnormalitis in MPS I, II ind IV, including thickening in the foveal area and thinning of parafoveal area [8]. However they found no retinal cheanges in MPS VI. Yoon et al. found that SD-OCT with ultrahigh resolution showed thinning due to degeneration of photoreceptor inner and outer (IS/OS), similar to atrophy of photoreceptors in patients with retinitis pigmentosa [22]. The varying degrees of thinning in MPS types are believed to result from different accumulated GAG products [17]. Our study confirms significant parafoveal and perifoveal retinal thinning in MPS especially MPS II. However, based on our methods, we cannot determine whether the thinning due to IS/OS degeneration or specific metabolic product accumulation.

The few previous studies on OCT-measured pRNFL thickness also revealed no significant difference between MPS patients and healthy subjects [5, 22]. However, our results indicate lower pRNFL in patients with optic atrophy on funduscopy. On average, the MPS types, except MPS II, showed a thickened pRNFL compared to the control group. Swelling of the optic disc may be secondary to GAG accumulation in the sclera and compression of the optic nerve and may also be responsible for increased IOP in some cases [6]. 

We observed a reduced and more irregular retinal capillary density by using OCT-A affecting MPS types I, II and IV. The mechanism inducing these changes is not known. Endothelial dysfuntcion associates with reduced NO synthesis has been described in MPS patients [25]. HMM^®^ was found to be unsuitable for examining MPS patients due to its complex handling requirements and the non-reproducibility of images obtained. To our knowledge, this is the first use of OCTA and HMM in MPS patients. We aimed to explore these techniques as new tools for future studies. OCTA allows qualitative assessment of vascular density by distinguishing blood flow from static tissue, while HMM offers detailed imaging of retinal structures, especially the photoreceptor mosaic. The focus of our study was to present qualitative imaging rather than quantitative results, allowing for a differentiated assessment of pathological changes that goes beyond purely metric data. Given the high interindividual variability of the measurements and potential influences of age or refractive status, a quantitative analysis seemed less informative. Instead, structural changes were qualitatively described using representative OCT scans. The aim was also to highlight clinically relevant imaging changes as they are assessed in routine ophthalmological evaluations. The presented OCT images thus reflect practical relevance without the need for extensive quantitative analysis. This study has several limitations. First, regional and ethnic variations in the prevalence of MPS resulted in an unequal distribution in MPS types in our sample. Additionally, physical and cognitive deficits in some MPS patients, particularly in those with MPS III, hindered or restricted some examinations. MPS patients exhibited a wide range of spherical refractive errors, from − 1.0 to + 17.0 diopters, with the majority being hyperopic and presenting with astigmatism. Matching the MPS and control groups with respect to refractive status was therefore challenging. This limitation should be taken into account when interpreting the OCT and OCT-A findings. The limited availability of imaging data in MPS patients resulted in a smaller sample size, which may have reduced the statistical power of the analysis and introduced potential bias. Nevertheless, several significant differences were observed between the MPS and control groups. The impact of the unequal group sizes should be considered when interpreting these results, particularly in the context of more advanced disease. Visual acuity testing is subjective, with a potential guessing bias and depended on patient compliance [26]. The quality of some OCT images was compromised, with inaccurate delineation of retinal boundaries necessitating retrospective editing, as noted in previous studies [10]. In the present study, the entire thickness of the retina was recorded instead of a differentiated analysis of the individual layers. Although this methodology significantly limits comparability with other studies, it offers a new basis for future investigations, particularly in MPS patients where precise detection using OCT, especially in MPS III, is difficult.

This study highlights significant posterior segment alterations in MPS patients. GAG accumulation in MPS can lead to reduced visual acuity, increased IOP, concentric visual field defects, retinal thinning, and vascular changes. These findings emphasize the importance of additional posterior segment evaluations, particularly for MPS types I, II, and III.

## Data Availability

The data collected and analyzed in the study are available on request from the corresponding author.
